# Pyrazine-2(1*H*)-thione

**DOI:** 10.1107/S2414314621011020

**Published:** 2021-10-29

**Authors:** Adrian Olszewski, Kinga Wzgarda-Raj

**Affiliations:** a Department of Physical Chemistry, Faculty of Chemistry, University of Lodz, Pomorska 163/165, 90-236 Lodz, Poland; Universidad de Los Andes, Venezuela

**Keywords:** crystal structure, 2-mercapto­pyrazine, hydrogen bonds, Hirshfeld surface analysis

## Abstract

The title compound, C_4_H_4_N_2_S, was obtained by the reduction of 2-mercapto­pyrazine (during its crystallization with 2-mercapto­pyrazine and isonicotinic acid *N*-oxide in ethanol solution. It crystallizes in the monoclinic space group P*2_1_/m*. In the crystal, the mol­ecules are linked by N—H⋯N and C—H⋯S hydrogen bonds.

## Abstract 2

In the crystal, the mol­ecules are linked by N—H⋯N and C—H⋯S hydrogen bonds.

## Graphical abstract

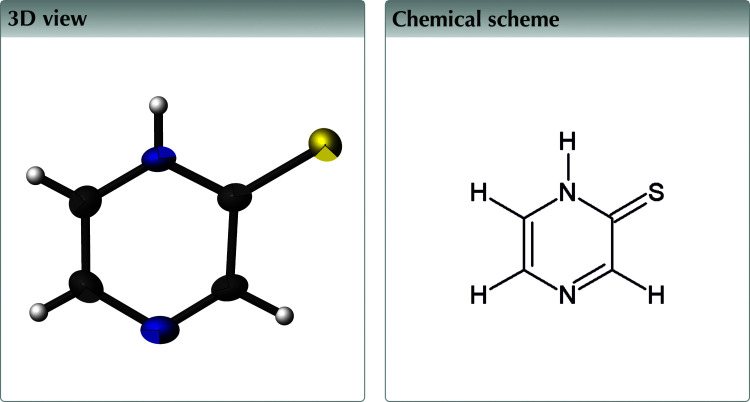

## Structure description

Pyrazine is an aromatic six-membered hetrocyclic that contains two nitro­gen atoms in positions 1 and 4. As a result, pyrazine has weaker basic properties than pyridine, pyridazine and pyrimidine. Pyrazine derivatives play an important role in chemotherapy (Wu *et al.*, 2012 ▸; Polshettiwar & Varma 2008 ▸; Goya *et al.*, 1997 ▸). Its derivatives possess diverse biological activities such as anti­diabetic, diuretic (Pranab *et al.*, 2011 ▸), anti-inflammatory (Chandrakant & Naresh, 2004 ▸), anti­microbial (Mallesha & Mohana 2011 ▸), analgesic (Doležal *et al.*, 2007 ▸) and anti­cancer (Kayagil & Demirayak, 2011 ▸). In addition, 2-mercaptopyrinosine derivatives are known to be cancer inhibitors (Mallesha & Mohana, 2011 ▸; Bonde & Gaikwad, 2004 ▸).

**Figure 1:**
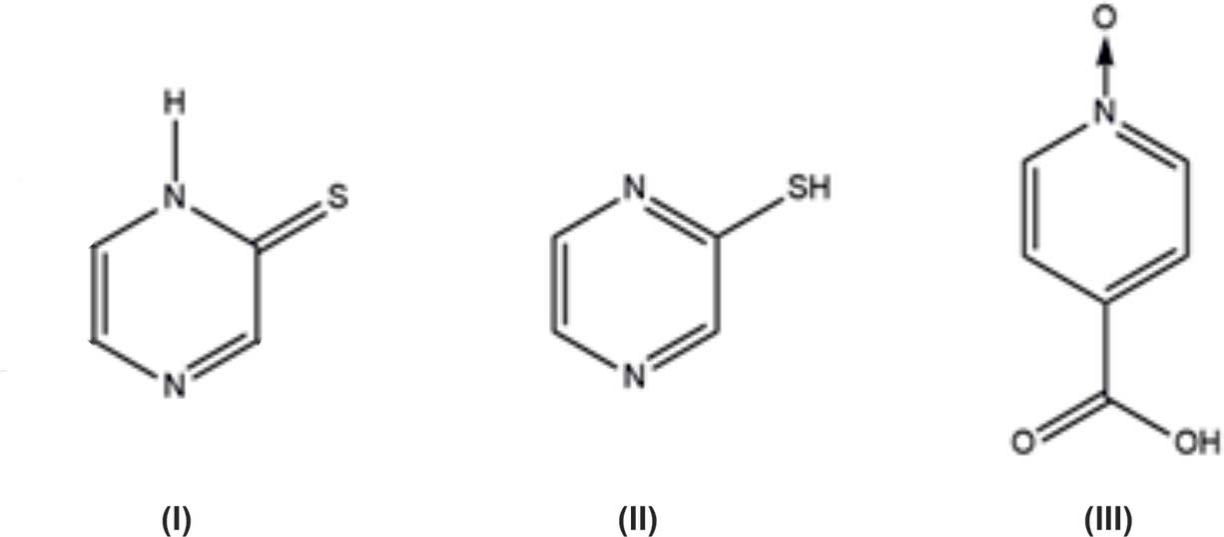
. Mol­ecular formulae of pyrazine-2(1*H*)-thione (I), 2-mercapto­pyrazine (II) and isonicotinic acid *N*-oxide (III).

**Figure 2:**
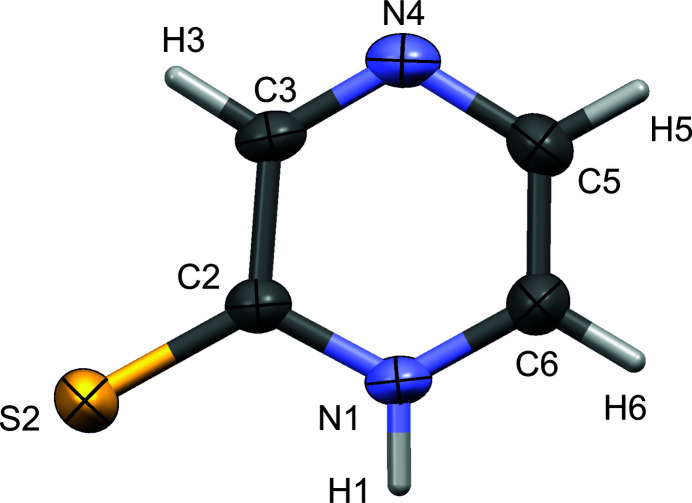
The mol­ecular structure of pyrazine-2(1*H*)-thione, showing the atom-labelling scheme and displacement ellipsoids at the 50% probability level.

The title compound pyrazine-2(1*H*)-thione (I) was obtained as a yellow solid by reduction of 2-mercapto­pyrazine (II) during its crystallization with 2-mercapto­pyrazine (II) and isonicotinic acid *N*-oxide (III) in ethanol solution (Fig. 1 ▸). Pyrazine-2(1*H*)-thione crystallizes in the monoclinic space group *P*2_1_/m. The atomic labelling scheme is shown in Fig. 2 ▸. In pyrazine-2(1*H*)-thione, being a reduced form of (II), there is one hydrogen atom at atom N1.

The C—C bond lengths are within the expected values known for aromatic systems. The N1—C2 and N1—C6 bond lengths [1.354 (3) and 1.355 (3) Å, respectively] are longer than those for N4—C3 and N4—C5 [1.299 (3) Å and 1.366 (3) Å)], respectively. This is the result of the protonation of the N1 atom. The C2—S2 bond length [1.671 (2) Å] is comparable within the 3σ criterion. All of the angles have usual values.

**Figure 3:**
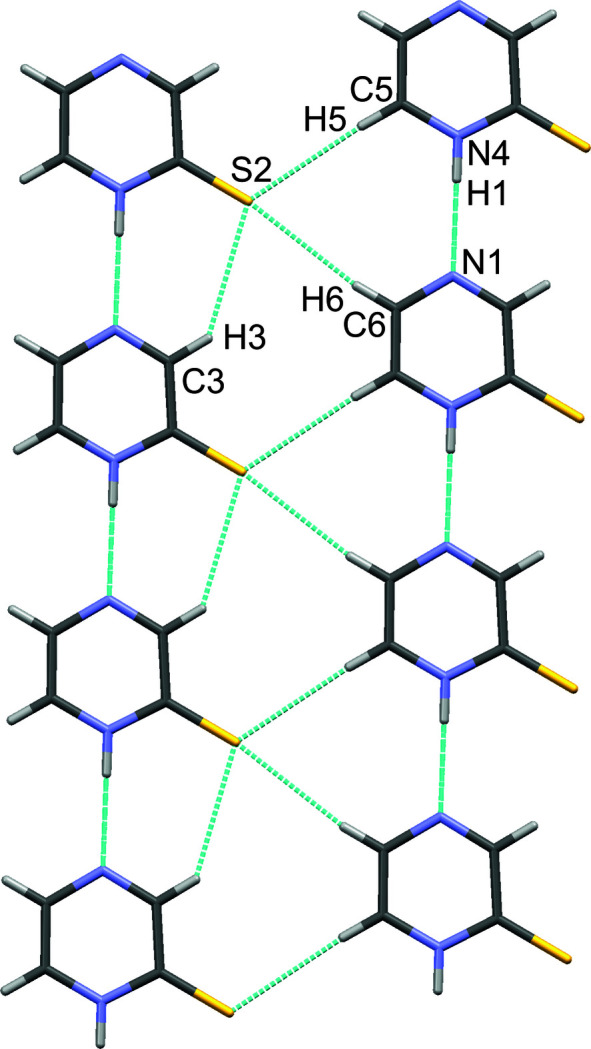
N–H⋯N and C—H⋯S hydrogen bonds between adjacent pyrazine-2(1*H*)-thione mol­ecules.

The crystal packing of pyrazine-2(1*H*)-thione is determined by hydrogen bonds of the N—H⋯N and C—H⋯S type (Table 1 ▸). Firstly, N1—H1⋯N4 hydrogen bonds [C⋯S = 2.893 (2) Å] between neighbouring mol­ecules form a chain. As a result, the mol­ecules are ordered along the [100] direction. This parallel arrangement is additionally stabilized by further inter­actions between adjacent mol­ecules [C3—H3⋯S2 = 3.716 (2) Å, C5—H5⋯S2 = 3.797 (3) Å and C6—H6⋯S2 = 3.775 (3) Å], as shown in Fig. 3 ▸.

**Figure 4:**
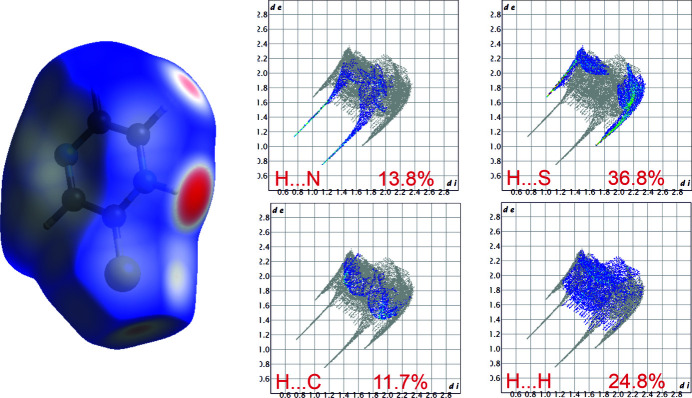
The mol­ecular Hirshfeld surfaces of pyrazine-2(1*H*)-thione mapped with *d*_norm_. Red areas represent inter­molecular contacts of distances shorter than the van der Waals separation.

Mol­ecular Hirshfeld surface (Spackman & Jayatilaka, 2009 ▸) and fingerprint plots (Spackman & McKinnon, 2002 ▸), were generated with *Crystal Explorer 3.1* (Wolff *et al.*, 2012 ▸) using the automatic procedures implemented in the program. The surfaces are mapped with a normalized contact distance (*d*_norm_), with values ranging from −0.58 to 1.05 a.u. Graphical representations of the Hirshfeld fingerprint plots for selected types of inter­molecular inter­actions are presented in Fig. 4 ▸. The C—H⋯S and N—H⋯N hydrogen bonds make major contribution to the overall Hirshfeld surface with 36.8% and 13.8% contributions, respectively. In addition, H⋯H (24.8%) and H⋯C (11.7%) contacts make a significant contribution to the crystal packing.

A search of the Cambridge Structural Database (CSD version 5.41, November 2019; Groom *et al.*, 2016) for 2-mercapto­pyrazine with no disorder, no other errors and only organic compounds yielded 79 structures. However, the structure of this compound and its oxidised form were not found.

## Synthesis and crystallization

Crystals suitable for *X*-ray measurements were obtained from commercially available reagents (Aldrich Chemical Co.) which were used without further purification. 0.5 mmol of 2-mercapto­pyrazine (II) was mixed with 0.5 mmol of iso­nico­tinic acid *N*-oxide (III) and dissolved in ethanol (4 ml). The obtained solution was kept at room temperature. Crystals (yellow plates) for *X*-ray diffraction were obtained after slow evaporation of the solvent within 2 weeks.

## Refinement

Crystal data, data collection and structure refinement details are summarized in Table 2 ▸.

## Supplementary Material

Crystal structure: contains datablock(s) I. DOI: 10.1107/S2414314621011020/dj4001sup1.cif

Structure factors: contains datablock(s) I. DOI: 10.1107/S2414314621011020/dj4001Isup2.hkl

CCDC reference: 2117037

Additional supporting information:  crystallographic information; 3D view; checkCIF report

## Figures and Tables

**Table 1:** Hydrogen-bond geometry (Å, °)

*D*—H⋯*A*	*D*—H	H⋯*A*	*D*⋯*A*	*D*—H⋯*A*
C5—H5⋯S2^i^	0.86 (3)	2.94 (3)	3.797 (3)	171 (3)
C6—H6⋯S2^ii^	0.90 (3)	2.88 (3)	3.775 (2)	173 (2)
C3—H3⋯S2^iii^	0.99 (3)	2.98 (3)	3.716 (2)	132 (2)
N1—H1⋯N4^iv^	0.85 (4)	2.04 (4)	2.893 (3)	178 (3)
C5—H5⋯S2^i^	0.86 (3)	2.94 (3)	3.797 (3)	171 (3)
C6—H6⋯S2^ii^	0.90 (3)	2.88 (3)	3.775 (2)	173 (2)
C3—H3⋯S2^iii^	0.99 (3)	2.98 (3)	3.716 (2)	132 (2)
N1—H1⋯N4^iv^	0.85 (4)	2.04 (4)	2.893 (3)	178 (3)

Symmetry codes: (i) 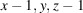 ; (ii) 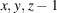 ; (iii) 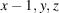 ; (iv) 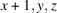 .

**Table 2:** Experimental details

Crystal data	
Chemical formula	C_4_H_4_N_2_S
*M*_r_	112.15
Crystal system, space group	Monoclinic, *P*2_1_/*m*
Temperature (K)	150
*a*, *b*, *c* (Å)	5.6113 (3), 6.4370 (6), 7.0923 (4)
β (°)	100.325 (6)
*V* (Å^3^)	252.03 (3)
*Z*	2
Radiation type	Mo *K*α
μ (mm^−1^)	0.49
Crystal size (mm)	0.18 × 0.06 × 0.04

Data collection	
Diffractometer	XtaLAB Synergy, Dualflex, HyPix
Absorption correction	Analytical (*CrysAlis PRO*; Rigaku OD, 2015 ▸)
*T*_min_, *T*_max_	0.991, 0.997
No. of measured, independent and observed [*I* > 2σ(*I*)] reflections	2812, 562, 496
*R*_int_	0.035
(sin θ/λ)_max_ (Å^−1^)	0.627

Refinement	
*R*[*F* ^2^ > 2σ(*F* ^2^)], *wR*(*F* ^2^), *S*	0.031, 0.088, 1.13
No. of reflections	562
No. of parameters	55
H-atom treatment	All H-atom parameters refined
Δρ_max_, Δρ_min_ (e Å^−3^)	0.24, −0.19

Computer programs: *CrysAlis PRO* (Rigaku OD, 2015 ▸), *SHELXT2018*/2 (Sheldrick, 2015*a* ▸), *SHELXL2018*/3 (Sheldrick, 2015*b* ▸) and *SHELXTL* (Sheldrick, 2008 ▸).

## full crystallographic data

### Crystal data

**Table:**

C_4_H_4_N_2_S	*F*(000) = 116
*M**_r_* = 112.15	*D*_x_ = 1.478 Mg m^−^^3^
Monoclinic, *P*2_1_/*m*	Mo *K*α radiation, λ = 0.71073 Å
*a* = 5.6113 (3) Å	Cell parameters from 1374 reflections
*b* = 6.4370 (6) Å	θ = 3.2–29.1°
*c* = 7.0923 (4) Å	µ = 0.49 mm^−^^1^
β = 100.325 (6)°	*T* = 150 K
*V* = 252.03 (3) Å^3^	Plate, yellow
*Z* = 2	0.18 × 0.06 × 0.04 mm

### Data collection

**Table:**

XtaLAB Synergy, Dualflex, HyPix diffractometer	496 reflections with *I* > 2σ(*I*)
Detector resolution: 10.4052 pixels mm^-1^	*R*_int_ = 0.035
ω scans	θ_max_ = 26.5°, θ_min_ = 2.9°
Absorption correction: analytical (CrysAlisPro; Rigaku OD, 2015)	*h* = −7→6
*T*_min_ = 0.991, *T*_max_ = 0.997	*k* = −7→8
2812 measured reflections	*l* = −8→8
562 independent reflections	

### Refinement

**Table:**

Refinement on *F*^2^	Primary atom site location: difference Fourier map
Least-squares matrix: full	Hydrogen site location: difference Fourier map
*R*[*F*^2^ > 2σ(*F*^2^)] = 0.031	All H-atom parameters refined
*wR*(*F*^2^) = 0.088	*w* = 1/[σ^2^(*F*_o_^2^) + (0.0411*P*)^2^ + 0.0767*P*] where *P* = (*F*_o_^2^ + 2*F*_c_^2^)/3
*S* = 1.13	(Δ/σ)_max_ < 0.001
562 reflections	Δρ_max_ = 0.24 e Å^−^^3^
55 parameters	Δρ_min_ = −0.19 e Å^−^^3^
0 restraints	

### Special details

**Table:**

Geometry. All esds (except the esd in the dihedral angle between two l.s. planes) are estimated using the full covariance matrix. The cell esds are taken into account individually in the estimation of esds in distances, angles and torsion angles; correlations between esds in cell parameters are only used when they are defined by crystal symmetry. An approximate (isotropic) treatment of cell esds is used for estimating esds involving l.s. planes.
Refinement. Hydrogen atoms of aromatic rings were introduced in calculated positions with idealized geometry and constrained using a rigid body model with isotropic displacement parameters equal to 1.2 the equivalent displacement parameters of the parent atoms. The H atom of the NH group was located in a difference Fourier map and freely refined.

### Fractional atomic coordinates and isotropic or equivalent isotropic displacement parameters (Å^2^)

**Table:**

	*x*	*y*	*z*	*U*_iso_*/*U*_eq_	
S2	0.96392 (11)	0.750000	0.80505 (9)	0.0517 (3)	
N1	0.8466 (3)	0.750000	0.4242 (3)	0.0354 (5)	
N4	0.3609 (3)	0.750000	0.4191 (3)	0.0371 (5)	
C3	0.5132 (4)	0.750000	0.5802 (3)	0.0335 (5)	
C2	0.7722 (4)	0.750000	0.5956 (3)	0.0321 (5)	
C6	0.6909 (4)	0.750000	0.2544 (4)	0.0418 (6)	
C5	0.4506 (4)	0.750000	0.2523 (4)	0.0436 (6)	
H5	0.355 (5)	0.750000	0.143 (5)	0.051 (8)*	
H6	0.743 (5)	0.750000	0.142 (4)	0.047 (8)*	
H3	0.458 (5)	0.750000	0.704 (4)	0.037 (7)*	
H1	0.999 (7)	0.750000	0.426 (5)	0.069 (10)*	

### Atomic displacement parameters (Å^2^)

**Table:**

	*U*^11^	*U*^22^	*U*^33^	*U*^12^	*U*^13^	*U*^23^
S2	0.0290 (4)	0.0950 (6)	0.0300 (4)	0.000	0.0026 (2)	0.000
N1	0.0185 (9)	0.0561 (13)	0.0327 (10)	0.000	0.0074 (8)	0.000
N4	0.0215 (9)	0.0495 (12)	0.0409 (11)	0.000	0.0069 (8)	0.000
C3	0.0217 (10)	0.0436 (13)	0.0371 (12)	0.000	0.0108 (9)	0.000
C2	0.0231 (10)	0.0414 (13)	0.0326 (11)	0.000	0.0076 (9)	0.000
C6	0.0301 (12)	0.0672 (18)	0.0292 (12)	0.000	0.0085 (10)	0.000
C5	0.0270 (12)	0.0693 (18)	0.0323 (13)	0.000	−0.0008 (10)	0.000

### Geometric parameters (Å, º)

**Table:**

S2—C2	1.671 (2)	C3—C2	1.437 (3)
N1—C2	1.354 (3)	C3—H3	0.99 (3)
N1—C6	1.355 (3)	C6—C5	1.346 (3)
N1—H1	0.85 (4)	C6—H6	0.90 (3)
N4—C3	1.299 (3)	C5—H5	0.86 (3)
N4—C5	1.366 (3)		
			
C2—N1—C6	123.0 (2)	N1—C2—S2	123.03 (17)
C2—N1—H1	117 (2)	C3—C2—S2	123.25 (18)
C6—N1—H1	120 (2)	C5—C6—N1	119.6 (2)
C3—N4—C5	118.37 (19)	C5—C6—H6	118.2 (19)
N4—C3—C2	124.3 (2)	N1—C6—H6	122.2 (19)
N4—C3—H3	121.6 (15)	C6—C5—N4	121.0 (2)
C2—C3—H3	114.1 (15)	C6—C5—H5	118 (2)
N1—C2—C3	113.7 (2)	N4—C5—H5	121 (2)
			
C5—N4—C3—C2	0.000 (1)	N4—C3—C2—S2	180.000 (1)
C6—N1—C2—C3	0.000 (1)	C2—N1—C6—C5	0.000 (1)
C6—N1—C2—S2	180.000 (1)	N1—C6—C5—N4	0.000 (1)
N4—C3—C2—N1	0.000 (1)	C3—N4—C5—C6	0.000 (1)

### Hydrogen-bond geometry (Å, º)

**Table:**

*D*—H···*A*	*D*—H	H···*A*	*D*···*A*	*D*—H···*A*
C5—H5···S2^i^	0.86 (3)	2.94 (3)	3.797 (3)	171 (3)
C6—H6···S2^ii^	0.90 (3)	2.88 (3)	3.775 (2)	173 (2)
C3—H3···S2^iii^	0.99 (3)	2.98 (3)	3.716 (2)	132 (2)
N1—H1···N4^iv^	0.85 (4)	2.04 (4)	2.893 (3)	178 (3)
C5—H5···S2^i^	0.86 (3)	2.94 (3)	3.797 (3)	171 (3)
C6—H6···S2^ii^	0.90 (3)	2.88 (3)	3.775 (2)	173 (2)
C3—H3···S2^iii^	0.99 (3)	2.98 (3)	3.716 (2)	132 (2)
N1—H1···N4^iv^	0.85 (4)	2.04 (4)	2.893 (3)	178 (3)

Symmetry codes: (i) *x*−1, *y*, *z*−1; (ii) *x*, *y*, *z*−1; (iii) *x*−1, *y*, *z*; (iv) *x*+1, *y*, *z*.
